# Adaptive versus fixed designs in confirmatory clinical trials: centering the choice on ethics

**DOI:** 10.1177/17407745261468001

**Published:** 2026-08-04

**Authors:** Stef Baas, Lukas Pin, David S. Robertson, Frank Bretz, Hearn Jay Cho, Laura Flight, Rieke van der Graaf, Mackenzie Graham, Peter Jacko, Thomas Jaki, Jonathan Kimmelman, Andrea Manfrin, Richard Milne, Pavel Mozgunov, Philip Pallmann, Tom Parke, Mahesh K. B. Parmar, Vinciane Pirard, Kate Sahan, Jerome Amir Singh, Brian D. M. Tom, John C. Whittaker, Katie Wozniak, David Wright, Haiyan Zheng, Alex John London, Mark Sheehan, Sofía S. Villar

**Affiliations:** 1 MRC Biostatistics Unit, University of Cambridge, UK; 2 Advanced Quantitative Sciences, Novartis Pharma AG, Basel, Switzerland; 3 Medical University of Vienna, Center for Medical Data Science, Institute of Medical Statistics, Austria; 4 The Multiple Myeloma Research Foundation, Norwalk, CT, USA; 5 Tisch Cancer Institute, Icahn School of Medicine at Mount Sinai, New York, NY, USA; 6 National Institute for Health and Care Excellence, Manchester, UK; 7 Department of Bioethics and Health Humanities, University Medical Center Utrecht, The Netherlands; 8 Ethox Centre, Oxford Population Health, University of Oxford, UK; 9 Department of Management Science, Lancaster University, UK; 10 Department of Machine Learning and Data Science, University of Regensburg, Germany; 11 Department of Equity, Ethics and Policy, McGill University, Montreal, QC, Canada; 12 Medicines and Healthcare products Regulatory Agency, London, UK; 13 RAND Europe, Cambridge, UK; 14 Cambridge Public Health, University of Cambridge, UK; 15 Centre for Trials Research, Cardiff University, UK; 16 Berry Consultants UK Ltd, Abingdon, UK; 17 Institute of Clinical Trials and Methodology, MRC Clinical Trials Unit, University College London, UK; 18 Global Medical Affairs Rare Diseases, Sanofi Belgium SA, Machelen, Belgium; 19 NIHR Oxford Biomedical Research Centre, Oxford University Hospital Trust, UK; 20 Howard College School of Law, University of KwaZulu-Natal, Durban, South Africa; 21 Dalla Lana School of Public Health, University of Toronto, ON, Canada; 22 Respiratory and Immunology Biometrics and Statistical Innovation, BioPharmaceuticals R&D, AstraZeneca, Cambridge, UK; 23 Department of Mathematical Sciences, University of Bath, UK; 24 Philosophy Department, Carnegie Mellon University, Pittsburgh, PA, USA

**Keywords:** design of experiments, design uncertianty, trial integrity, bioethics, multi-stakeholder perspectives

## Abstract

**Background:**

Because confirmatory clinical trials are costly, large-scale endeavors, the choice of their design carries significant weight. While the current methodological landscape offers tools to address residual pre-trial uncertainty through prespecified adaptations to design elements, design choices remain frequently constrained by prevailing orthodoxies.

**Methods:**

We examine how unacknowledged design uncertainties (e.g., sample size calculations based on incorrect effect size or variance estimates) impact the statistical, ethical, and resource-stewardship goals of confirmatory trials. We contrast how fixed and adaptive design strategies handle these uncertainties, detailing the operational safeguards, firewalls, and trade-offs (e.g., complexity, operational bias) required to preserve trial integrity.

**Results:**

Strict adherence to default templates prevents stakeholders from matching the design strategy to the specific complexities of the research question. We observe that the gold standard status of fixed designs often obscures their limitations in handling design uncertainties. A rigid adherence to these conventions can ethically hinder a trial’s ability to deliver conclusive results. Conversely, while adaptive designs can offer tools to efficiently reduce uncertainty, we acknowledge that adaptive elements are not without cost; their implementation requires rigorous safeguards to manage specific risks to trial integrity and potential operational biases.

**Conclusion:**

To better align clinical research with its ethical and scientific mandates, we issue two calls to action. First, trial designers, including statisticians, must clearly articulate how target and design uncertainties impact ethical obligations, promoting an open-minded evaluation of both fixed and adaptive methods. Second, stakeholders must foreground ethical considerations during design selection, requiring explicit justification for the use—or non-use—of adaptive elements. The ethical path forward is not to default to the old or blindly adopt the new, but to explicitly justify the chosen design based on its responsiveness to the specific uncertainties of the trial. We posit that trial design must transition from a habit-based process to one where pre-trial uncertainties are openly discussed and addressed.

## Background

Clinical trials are tools for generating the evidence that guides critical decision-making across the entire healthcare landscape. This evidence is relied upon by everyone from patients and clinicians to policymakers and researchers, influencing individual health, the safety, equity, and efficiency of health systems, the use of scarce resources, and the direction and pace of innovation. Clinical trials are also social undertakings that must advance and respect inherently ethical purposes. As a result, their design must be responsive to a complex mix of scientific, statistical, and ethical requirements. In April 2025, a workshop in Cambridge, UK, convened stakeholders to consider how adaptive trial designs relate to these requirements. The workshop, with 113 participants, included methodological statisticians, research ethicists, clinical trialists (from both industry and academia), as well as representatives of regulators, health technology assessment bodies, patients, and the public. This article is a result of that workshop and subsequent discussions.

The interaction among these diverse stakeholders quickly surfaced two ethically relevant tensions. First, the task of ensuring that individual trials employ rigorous scientific, statistical, and ethical standards to advance clinical research objectives is hindered by persistent divergences in perspectives about what constitutes an adaptive design and by prevailing orthodoxies and heuristics regarding the merits of particular design elements. Second, many stakeholders do not appreciate the way that design uncertainty—understood as uncertainty about assumptions necessary to design a trial—can impact the statistical and ethical merits of a design. Specifically, trials are designed based on numerous assumptions—such as expected effect sizes or event rates—that are frequently uncertain or outright incorrect. These tensions also interact. As an example, the common heuristic of labeling certain design features as “gold standard” (such as a fixed 1:1 randomization ratio and a fixed set of interventions decided a priori) sets a default assumption in favor of fixed designs while obscuring the impact that incorrect design assumptions have on the scientific and ethical merits of those designs.

This article’s central claim is that stakeholders can better assess the merits of specific trials given the demands of the context by (1) foregrounding the fundamentally ethical considerations that shape both the objectives and the constraints of the trial, (2) requiring that the trial design be justified in terms of their responsiveness to these objectives, and (3) including consideration of the impact of design uncertainty on proposed designs. This claim is supported by four arguments. Together, this claim and its supporting arguments ground a call to action for all stakeholders in this enterprise to shift away from simple generalizations about the merits of design elements and to better connect design choices to the ethical objectives that determine their merits in particular contexts and that justify the conduct of a particular trial.

## The ethical pitfall of persistent divergence in perspectives

Clinical trials require the cooperation of diverse stakeholders who often differ in their disciplinary background, their familiarity with the state of the art in these various fields, their practices for using particular terms, or the lessons they draw from salient cases. Despite a growing literature in statistics,^1–8^ ethics,^9–15^ and regulatory guidance^16–19^ on adaptive trial designs, participants reflected lingering divergences in perspective also observed in the literature. Table S1 in the Supplemental Material provides specific examples from the literature to substantiate this claim. These variations and divergent perspectives complicate communication and the process of evaluating particular designs based on common ground. Two types of variation are of particular importance.

First, stakeholders often take the term “adaptive trial” to refer to different things. Some stakeholders lump together both prespecified and ad hoc adaptations under the same heading. Others take the term to refer to specific types of adaptation, such as response-adaptive randomization, and to exclude group sequential designs. Some stakeholders associate adaptive designs with Bayesian methods, while others recognize that this is not the case.

Second, these diverging uses of the term are often associated with differing normative attitudes. Some regard the term “adaptive” as purely descriptive. Some associate adaptive designs with the stigma from early controversies over the use of response-adaptive elements in the ECMO trial.^20^ Others embrace adaptive designs for virtues prominently displayed during the COVID-19 pandemic^21^ and the interest this spurred.^22,23^ Adaptive designs are frequently referred to as novel, reinforcing the status of fixed designs as “traditional” and the “gold standard.” In other cases, adaptive designs are associated with a particular type of ethical benefit, such as “individual ethics” understood as concern for promoting beneficial outcomes for trial participants, while fixed designs are associated with “collective ethics” understood as concern for the well-being of future patients.^24–26^

## Preferred definitions

To avoid confusion, the stakeholders in clinical research require definitions that are analytically sound and both statistically and ethically relevant. We use the term “adaptive design element” to refer to any prespecified, data-driven (meaning the data arising from the clinical trial itself) alteration to some aspect of a trial, paired with a prespecified design-based analysis. Adaptations can involve design elements such as the number of active interventions (i.e. dropping or adding arms), the procedure for participant allocation, sample size, trial population, or some aspect of the trial hypothesis based on accumulating trial data. This definition entails two broad contrast classes. A “fixed design element” refers to a feature of a trial that is determined at the design stage and remains unchanged throughout the trial. Both fixed and adaptive designs contrast with ad hoc alterations because they explicitly pair the trial structure with a prespecified statistical analysis plan. In contrast, ad hoc alterations are unplanned changes that lack this pre-defined framework. Consequently, they are typically subject to evaluation by an oversight committee to assess their validity and impact. Figure 1 clarifies these definitions, visually positioning ad hoc alterations in relation to both fixed and adaptive design elements. We define an “adaptive trial” as any trial that includes *at least one* adaptive design element and a corresponding prespecified design-based analysis. In contrast, a “fixed design” is a trial in which *all* design elements are determined at the design stage and whose analysis assumes they remain unchanged throughout the trial.^27^

**Figure 1.**
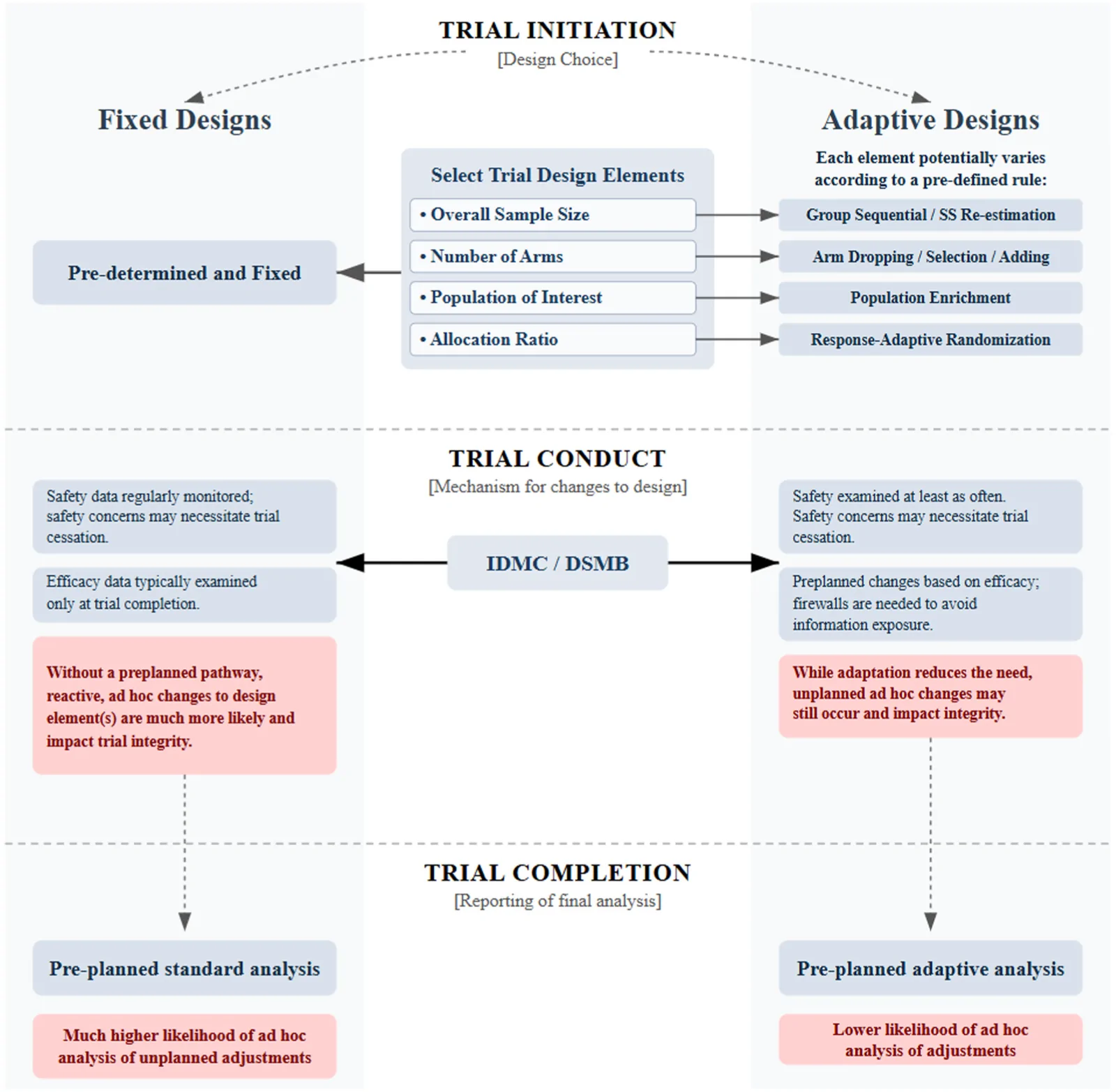
Key differences between *fixed* and adaptive designs. *Note*. The top part of Figure 1 lists four selected design elements that are predetermined and unchanged in a *fixed* randomized controlled trial design (left) and that can be altered according to prespecified, data-driven rules in adaptive designs (right). The middle part of Figure 1 indicates differences in the ways in which an independent data monitoring committee (IDMC)/data and safety monitoring board (DSMB) works under a *fixed* design and an adaptive design. The bottom part of Figure 1 highlights the differences in preplanned analysis at the end of each case.

These definitions have several virtues. First, it is important to distinguish preplanned design elements from ad hoc alterations because the latter are not *design* elements—they do not reflect an explicit scientific and statistical strategy for inquiry whose safeguards to protect trial integrity can be scrutinized by stakeholders before a study is initiated. Second, these definitions group aspects of trials together by features that relate directly to the statistical and ethical merits of a study, rather than by social convention. Third, they correct for misperceptions regarding the novelty of adaptive elements since the idea that data-driven, prespecified adaptive design elements might improve trial efficiency predates the first formal RCT by Bradford Hill in 1948.^28^ Finally, these definitions make it easier to highlight the way in which design uncertainty can impact the performance of trials that employ adaptive versus fixed design elements.

## Prioritizing ethical and statistical merit over convention in design choice

We claim that stakeholders can better coordinate to identify the best design in particular cases by foregrounding the ethical values at stake and then considering whether fixed or adaptive design elements best promote these values under the circumstances that are likely to obtain in actual practice.

For clarity, we divide the relevant ethical values into three categories. The first involves the core mission of clinical research, namely, generating the evidence needed to improve the safety, effectiveness, and equity of the care received by patients and the efficiency of the health systems that deliver it.^29^ These considerations together constitute the “social value” of the information generated in research.^30^ The second involves eliminating or reducing avoidable harms and burdens to participants, including harms from allocating more participants than needed to establish whether interventions are more (or less) beneficial than a feasible alternative. The third involves responsible stewardship of scarce resources, which includes clinical personnel and the financial costs associated with running a trial.

Articulating the likely circumstances under which a design will be implemented requires explicit consideration of uncertainty around assumptions required for such a trial to advance these ethical goals. This includes elaborating the conditions under which fixed or adaptive design elements are feasible and the ease or difficulty of implementing them adequately in practice. For example, feasibility requires the primary outcome of adaptation to be observable in enough time to allow for the design modification,^31^ while maintaining integrity during correct implementation often requires stringent firewalls and procedures when data are analyzed more often, such as in frequent interim analyses. The absence or lowered quality of these firewalls inherently poses a heightened risk to trial integrity—a risk less likely to occur in a non-adaptive, fixed design.

Four arguments support our claim.

First, there are circumstances in which fixed designs fail to advance any of the ethical values outlined here, and this outcome could be avoided with the careful use of adaptive design elements. These circumstances arise when trials fail to produce information that answers their motivating question due to faulty assumptions about an important design element.^32^ Such trials lack social value, subject trial participants to unnecessary risk and burden, and waste the time and resources of stakeholders. A recent review of Cochrane meta-analyses^33^ indicates that “underpowered studies made up the entirety of the evidence in most Cochrane reviews.” While the specific causes of this underpowering vary and we cannot precisely quantify the proportion of these trials that were intended as confirmatory, this serves as a proxy for the magnitude of the problem. When this problem is driven by uncertainty in pre-trial assumptions, an adaptive design becomes a valuable choice.

For a fixed design to advance these three ethical objectives, it requires that no circumstances—and in particular no circumstances relating to data emerging from the trial itself—arise that necessitate major changes to the trial. However, such circumstances are common, and a weakness of fixed designs is that they do not specify in advance how to respond to potentially foreseeable eventualities in ways that address each of these ethical concerns in a principled way (Figures 1 and 2).

**Figure 2.**
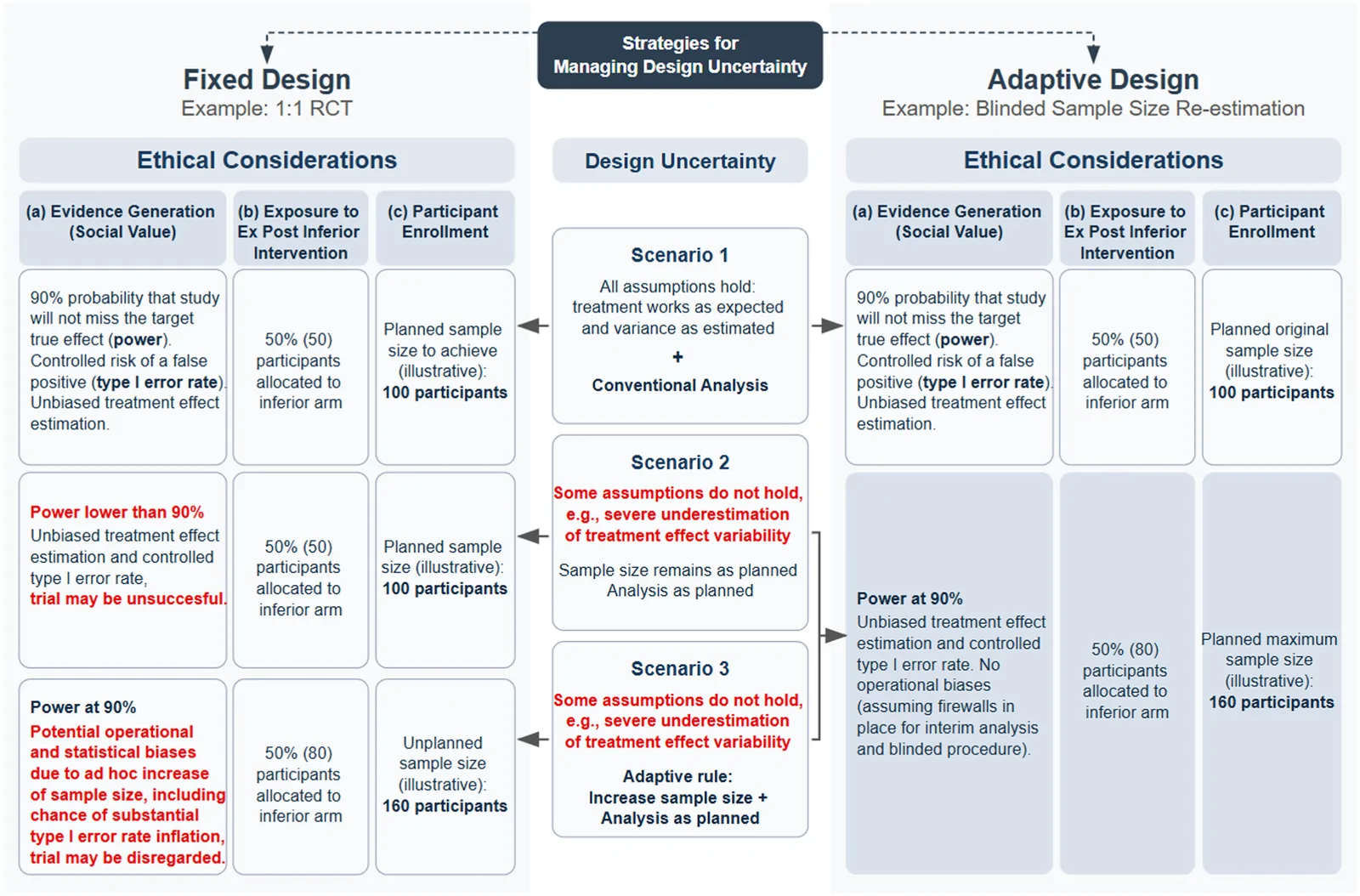
Conceptual comparison of the ethical considerations of *fixed* versus adaptive designs for some scenarios. *Note*. This schematic provides a conceptual overview of how a *fixed* design compares with sample size re-estimation (a type of adaptive design) under design uncertainty, in terms of the ethical dimensions of preserving social value (Column (a)), exposure to the ex post inferior intervention (i.e. harm to participants, Column (b)), and stewarding scarce resources (participant enrollment, Column (c)). For clarity, we assume normally distributed data and a blinded sample size review procedure after a *fixed* sample size of participant data is collected. Operational bias refers to the unintentional influence on the outcome of a clinical trial by unblinded analysts having access to interim data.

For example, to determine the necessary sample size of a trial, researchers must estimate the variance of the treatment effect estimator of interest to ensure sufficient power to detect a true meaningful difference of interest. Figure 2 provides a step-by-step illustrative comparison of how this requirement is met (or failed) under fixed versus adaptive designs. In a fixed design, researchers typically use an estimate of this variance, determine the total size of the trial, and run it to completion. However, despite a well-justified choice of an estimate, if this is lower than the true variance (as suggested by the trial data), maintaining the plan can result in an underpowered trial, reducing social value and potentially requiring additional studies to answer the trial question.^33^ An adaptive strategy like sample size re-estimation can better respond to this uncertainty by adding prespecified interim looks at the accumulating trial data, for example, at a point chosen to coincide with the initially planned total sample size of a fixed design. At this point, the trial data are analyzed so that researchers can obtain a more accurate estimate of the variance and adjust the final sample size accordingly while ensuring that the desired statistical power is maintained. However, this flexibility is not without risk.^34^ A crucial part of this consideration is determining the necessary level of unblinding required to execute sample size re-estimation with minimal risks to the trial integrity.

An advantage of adaptive designs is that they use predetermined strategies to respond to uncertainty around crucial assumptions by updating estimates as data accumulate and, if necessary, altering the original design’s elements. When studies (fixed or adaptive) lack adequate power, error rate control, bias mitigation, or suffer from other methodological shortcomings, they can expose participants to the cost, inconvenience, risk, and harms associated with trial participation without producing the social value that was regarded as necessary to justify those burdens at the time the trial was designed. This was illustrated during the COVID-19 pandemic, where the proliferation of small, uncoordinated studies produced redundant efforts, wasted resources, and imposed unredeemed burdens on participants.^35,36^

The upshot of our first argument is that some trials fail to produce socially and scientifically valuable information because they are based on faulty design assumptions such that the use of adaptive alternatives could have avoided this outcome. Consequently, investigators may resort to ad hoc modifications to rescue the trial, thereby introducing risks to integrity that a planned adaptive design could have better controlled. Stakeholders will be better able to advance the social and scientific purposes of confirmatory trials if they routinely consider the merits of adaptive designs as a strategy to address design uncertainty.

Second, at the design stage, every trial requires that the ethical values articulated here be integrated or balanced appropriately. Adaptive designs can make it easier to remain faithful to that balancing once a design is implemented. For example, ethical research begins in a state of uncertainty about the relative clinical merits of a set of interventions, and each trial must specify the features of the evidence (e.g. power to detect an effect of a certain size) that will count as a sufficient reduction in uncertainty ex post.^37^ Prespecified stopping rules enable stakeholders to articulate precise conditions under which evidence of futility or benefit that arises during the conduct of the trial is sufficiently strong that continuing with the initial randomization scheme would intentionally subject some trial participants to harms that are now avoidable, given the weight of accumulating evidence in the trial. Stopping early can avert unnecessary harms and the expenditure of resources that are unlikely to be redeemed by the social value of the information generated. Although early stopping can reduce the precision of effect estimates or introduce bias,^38,39^ preplanning enables stakeholders to anticipate and manage these issues within defined criteria, potentially making the primary trial result more credible and action-guiding.^40,41^ Stopping studies in the absence of prespecified stopping rules can delay the stopping time, introduce bias, and lead to type I error rate inflation. Some avoidable harms related to safety can be averted through the use of independent data monitoring committees. If the underlying trial design is fixed, independent data monitoring committees must make ad hoc judgments about efficacy or the tradeoffs between participant burden and benefit (see middle part of Figure 1).

Third, our claim can encourage stakeholders to consider whether these ethical values can be better advanced by increased coordination and planning not just within individual studies, but across “trajectories” of inquiry. During the COVID-19 pandemic, global platform trials like RECOVERY^42^ and SOLIDARITY^43^ examined numerous interventions, delivering rapid, reliable answers. Master protocols require stakeholders to consider not just the design of individual trials, but the design of sets or “trajectories” of trials and can fruitfully employ adaptive elements, for example, to prospectively determine when to add or drop arms or to dynamically adjust sample size. These strategies can reduce the time between trial phases and the time and effort expended on independent review of individual trials. Enforcing a common statistical framework on “trajectories” of trials can also increase the bandwidth of information produced and promote interoperability.^44^ In the context of rare diseases, employing such a common statistical framework can permit the testing of more interventions with fewer participants than uncoordinated pairwise trials.

Finally, by asking stakeholders to evaluate the relative merits of alternative designs in relation to these core ethical values, our claim centers on reasoned assessments and the ethical grounds of designs. This focus provides a counterweight to fads, stigma, uncritical adherence to convention, and advocacy for particular designs or statistical traditions (such as Bayesian or frequentist schools). We have tried to illustrate how adaptive designs can better advance these goals in some cases while recognizing that adaptive designs do not advance these goals in other cases. Some adaptations can introduce internal validity threats that must be stringently addressed and managed. Other adaptations can tax the ability of downstream evidence users to formulate independent interpretations of trial results. Adaptations motivated by a sponsor’s goals may cut short data collection that other stakeholders may need. This includes statistical reasons to avoid them, such as the possibility of substantial temporal trends that would be difficult to handle with a prespecified analysis plan.^45,46^ In addition, adaptive designs often require increasing complexity that can challenge the ability of stakeholders to comprehend the cumulative effects of the design choices on the dimensions outlined here.^15,47^ Here again, whether the benefits derived from increased complexity are sufficient or insufficient to justify using an adaptive design requires careful assessment of the relevant tradeoffs. More complex designs can also make higher demands on infrastructure, from computing capacity to the availability of experienced statisticians and the capacity of various review committees to oversee their conduct. Whether these hurdles are surmountable depends on the types of adaptations contemplated and the level of resources and infrastructure available. The proliferation of more complex designs has the potential to worsen health research disparities. Stakeholders from low- and middle-income countries and other under-resourced settings should consider these practicalities and balance the added complexities and validity threats for adaptive designs against their capacity to respond to design uncertainty using fixed designs.

Ensuring that stakeholders, across the full range of global settings, understand the benefits and limits of designs, adaptive and non-adaptive, and the conditions under which they best realize these ethical advantages is necessary to ensure that every trial design is justified by the requirements of the clinical trial and the ethical and methodological merits of the approach taken. There is (ethical) work for stakeholders in this enterprise to do to remove systemic barriers to ensure equitable consideration of design options, to foster awareness of design uncertainty, and the relative merits and challenges associated with fixed and adaptive elements for delivering high-quality evidence that advances the ethical objectives elaborated here. Some of this work has begun and is in development (see Table 1 for explicit pairings of trials and methods). For example, the FDA Complex Innovative Trial Design program^48^ and recent regulatory guidance on adaptive designs such as ICH E20^18^ represent a movement in the right direction. However, given the diversity of stakeholders and the challenges of communicating across disciplinary boundaries, we need imaginative strategies for fostering reasoned evaluations. This is not just about the dissemination of information but requires active involvement with stakeholders, including patients and the public. One of the key learnings from our work with patient and public contributors was about the need for engagement strategies which go beyond lectures and training courses and include bespoke approaches that give stakeholders direct exposure to ethical considerations surrounding the use of these techniques and how to think through their application in real cases.

**Table 1.** Adaptive trial elements: methodological concepts and illustrative trial examples.

Adaptive element	References to trial examples, documentation, and treated diseases/disorders	References to methodological papers/books
Sample size re-estimation	AIMM^49^ (metabolic disorders); NOTACS^50^ (respiratory complications after heart surgery)	Proschan;^1^ Pritchett et al.^2^
Group sequential design	PROTECTION^51^ (acute kidney injury)	Jennison and Turnbull^3^
Adding/dropping/selecting treatment arms	RECOVERY^42^ (COVID-19); STAMPEDE^52^,^53^ (cancer)	Greenstreet et al.;^4^ Choodari-Oskooei et al.^5^
Population selection (adaptive enrichment designs)	TAPPAS^54^ (cancer)	Simon and Simon;^6^ Thall^7^
Response-adaptive randomization	endTB^55^ (tuberculosis)	Robertson et al.^8^

*Note*. This table lists several adaptive elements (Column 1), gives references of trials (as well as disease/disorder areas) where the design included said elements (Column 2), and provides methodological references (Column 3).

## Conclusion

Stakeholders in clinical research require a framework for evaluating clinical trial designs that fosters an open-minded evaluation of the relative merits of fixed versus adaptive design elements. We have argued that this might be achieved by foregrounding fundamentally ethical considerations. That is, through proactive consideration of design uncertainty and whether fixed or prespecified, data-driven adaptations best promote social value, reduce harms to participants, and make effective use of scarce resources. This shift in perspective would enable a more explicit evaluation of the potential benefits of adaptive methodologies (where feasible) and facilitate their adoption where they serve these ethical objectives through a rigorous system of operational safeguards. These arguments have important practical consequences for stakeholders, particularly methodologists and regulators, by encouraging a pathway for this change across the clinical trials landscape.

## Supplemental Material

sj-docx-1-ctj-10.1177_17407745261468001 – Supplemental material for Adaptive versus fixed designs in confirmatory clinical trials: centering the choice on ethics Supplemental material, sj-docx-1-ctj-10.1177_17407745261468001 for Adaptive versus fixed designs in confirmatory clinical trials: centering the choice on ethics by Stef Baas, Lukas Pin, David S. Robertson, Frank Bretz, Hearn Jay Cho, Laura Flight, Rieke van der Graaf, Mackenzie Graham, Peter Jacko, Thomas Jaki, Jonathan Kimmelman, Andrea Manfrin, Richard Milne, Pavel Mozgunov, Philip Pallmann, Tom Parke, Mahesh K. B. Parmar, Vinciane Pirard, Kate Sahan, Jerome Amir Singh, Brian D. M. Tom, John C. Whittaker, Katie Wozniak, David Wright, Haiyan Zheng, Alex John London, Mark Sheehan and Sofía S. Villar in Clinical Trials
