# Supplementary material for: Adaptive versus fixed designs in confirmatory clinical trials: centering the choice on ethics
Source: Clin Trials. 2026 Aug 4;23(5):472–81. doi: 10.1177/17407745261468001 (PMC13624349; doi:10.1177/17407745261468001)
Supplement: Supplementary file 1 [file sj-docx-1-ctj-10.1177_17407745261468001.docx]

**Table S1: Examples of persistent differences in perspective on adaptive design elements and adaptive trials.**

| The gold standard | "Fixed randomized clinical trials (RCTs) are widely considered the gold standard for determining the relative efficacy of medical treatments.” ([1](https://doi.org/10.1186/s12910-015-0022-z))  "Fixed randomized clinical trials are considered the current gold standard for evaluating the efficacy of novel treatments, where the anticipated effect size and the estimated event rate in the control group are used to determine a fixed sample size.” ([2](https://doi.org/10.1186/s13063-020-04466-0))  "Fixed randomized controlled trials remain the gold standard in clinical drug development; however, there is growing interest in innovative trial designs with the potential to increase the efficiency of the development process.” ([3](https://www.brookings.edu/wp-content/uploads/2014/03/Discussion-Guide.pdf))  "EFPIA acknowledges that the traditional RCT is the current gold standard for providing robust evidence to support an evaluation of efficacy and safety leading to the registration of a medicinal product.” ([4](https://www.efpia.eu/media/guokpw0b/an-efpia-position-paper-on-randomised-pragmatic-trials-to-generate-high-quality-real-world-evidence-for-regulatory-decisions.pdf)) |
| --- | --- |
| Adaptive designs and novelty | “Although adaptive designs have been the focus of continuing research and debate in recent years, they have never become part of mainstream clinical research methodology” ([5](https://doi.org/10.1016/S0197-2456(01)00122-2))  Authors searched for adaptive designs in all trials on ClinicalTrials.gov from Jan 2006 to July 2021 and only found <300 examples ([6](https://doi.org/10.12793/tcp.2023.31.e21)) |
| Ad hoc adaptations included as an “adaptive” element or adaptive trial. | “an adaptive design that falls into a gray area with respect to whether or not it meets the FDA definition of being pre-specified” ([7](https://doi.org/10.1177/1740774516643689))  Unplanned arm adding ([8](https://doi.org/10.1136/gut.2010.221127))  Unplanned sample size increase ([9](https://www.ncbi.nlm.nih.gov/books/NBK604459/))  "The DSMB is in a tempting position to be able to implement adaptive trial methods after the examination of interim data. This currently runs specifically against the regulatory concepts of adaptive trial designs, in which adaptive designs must be prespecified before an interim analysis, as the FDA draft guidance for adaptive trial designs describes, but might be permissible under the independent oversight of a DSMB. The FDA does recognize that during the course of a trial, outside information may become available that suggests that trial changes should be made to protect patient safety. FDA guidance comments that in such a case, an adaptive implementation may be warranted, and the sponsor should still remain blinded to the interim results. The DSMB may thus be the best positioned to help with a conversion to an adaptive design." ([10](https://doi.org/10.1016/j.jacbts.2021.09.005)) |
| Uncertainty about whether group sequential designs are adaptive designs | “In the beginning, the already well‐established group sequential community was reluctant to accept connections between adaptive and group sequential designs. There was a sharp controversial discussion on the scientific value of applying adaptive designs in contrast to group sequential designs [14](https://pmc.ncbi.nlm.nih.gov/articles/PMC6680191/#sim6472-bib-0014), [15](https://pmc.ncbi.nlm.nih.gov/articles/PMC6680191/#sim6472-bib-0015), [16](https://pmc.ncbi.nlm.nih.gov/articles/PMC6680191/#sim6472-bib-0016), [17](https://pmc.ncbi.nlm.nih.gov/articles/PMC6680191/#sim6472-bib-0017), [18](https://pmc.ncbi.nlm.nih.gov/articles/PMC6680191/#sim6472-bib-0018) mainly because of the violation of the sufficiency principle [19](https://pmc.ncbi.nlm.nih.gov/articles/PMC6680191/#sim6472-bib-0019). Moreover, this critical view stated that classical group sequential designs are flexible and adaptive enough, in the sense that the sample size is changed in a data‐driven way through the introduction of stopping rules. Interestingly, although the arguments have not changed, some of the authors having been very critical initially are nowadays accepting adaptive designs. Today, it is commonly accepted that group sequential trials can be considered as a special case of the more general adaptive designs” ([11](https://doi.org/10.1002/sim.6472))  “We do not regard [group sequential designs as] adaptive designs here because neither involves modifying a study element and continuing the trial with the revised methods.” ([12](https://doi.org/10.1093/cid/cix907))  “Trials with traditional (or classical) group sequential design are not allowed to modify the pre-specified study criteria, such as sample size, frequency of interim analyses, length of study and trial stopping rule [[33](https://bmcmedresmethodol.biomedcentral.com/articles/10.1186/s12874-023-02024-1#ref-CR33)]. Because of this, in this review this design is not considered to be an adaptive design.” ([13](https://doi.org/10.1186/s12874-023-02024-1))  “There are a multitude of adaptive designs, with the most common including group sequential designs, adaptive randomization, pick-the-winner/drop-the-loser, sample size reestimation, adaptive enrichment, and Bayesian adaptive designs” ([14](https://journals.lww.com/pccmjournal/fulltext/2023/09000/adaptive_clinical_trials_in_pediatric_critical.4.aspx)) |
